# Maximum Entropy Models for Fatigue Damage in Metals with Application to Low-Cycle Fatigue of Aluminum 2024-T351

**DOI:** 10.3390/e21100967

**Published:** 2019-10-03

**Authors:** Colin Young, Ganesh Subbarayan

**Affiliations:** 1 School of Mechanical Engineering, Purdue University, West Lafayette, IN 47907-2088, USA; cyoung8@ford.com; 2 Ford Corporation, Dearborn, MI 48124, USA

**Keywords:** MaxEnt distributions, fatigue damage, low-cycle fatigue, thermodynamic entropy

## Abstract

In the present work, we propose using the cumulative distribution functions derived from maximum entropy formalisms, utilizing thermodynamic entropy as a measure of damage to fit the low-cycle fatigue data of metals. The thermodynamic entropy is measured from hysteresis loops of cyclic tension–compression fatigue tests on aluminum 2024-T351. The plastic dissipation per cyclic reversal is estimated from Ramberg–Osgood constitutive model fits to the hysteresis loops and correlated to experimentally measured average damage per reversal. The developed damage models are shown to more accurately and consistently describe fatigue life than several alternative damage models, including the Weibull distribution function and the Coffin–Manson relation. The formalism is founded on treating the failure process as a consequence of the increase in the entropy of the material due to plastic deformation. This argument leads to using inelastic dissipation as the independent variable for predicting low-cycle fatigue damage, rather than the more commonly used plastic strain. The entropy of the microstructural state of the material is modeled by statistical cumulative distribution functions, following examples in recent literature. We demonstrate the utility of a broader class of maximum entropy statistical distributions, including the truncated exponential and the truncated normal distribution. Not only are these functions demonstrated to have the necessary qualitative features to model damage, but they are also shown to capture the random nature of damage processes with greater fidelity.

## 1. Introduction

The wrought aluminum alloy 2024-T351 is an important light structural metal commonly used in aerospace and other weight-critical applications [1]. A common approach to modeling the low-cycle fatigue (LCF) life of this material and many other metals is the Coffin–Manson relationship [1,2]:

(1) $$\frac{\Delta \epsilon_{p}}{2}=\epsilon_{f}\prime {\left(2N_{f}\right)}^{c}$$

This equation is intended to cover the range of life from 1 to about 20,000 reversals, where macroscopic plastic strain is measurable. However, as has been pointed in the literature [2], Equation (1) is less successful in fitting data in the very low reversal count range of 1 to about 200. The inadequacy of Equation (1) for modeling a representative LCF data set for 2024-T351 is demonstrated below and motivates an alternative LCF modeling approach.

**Figure 1:**
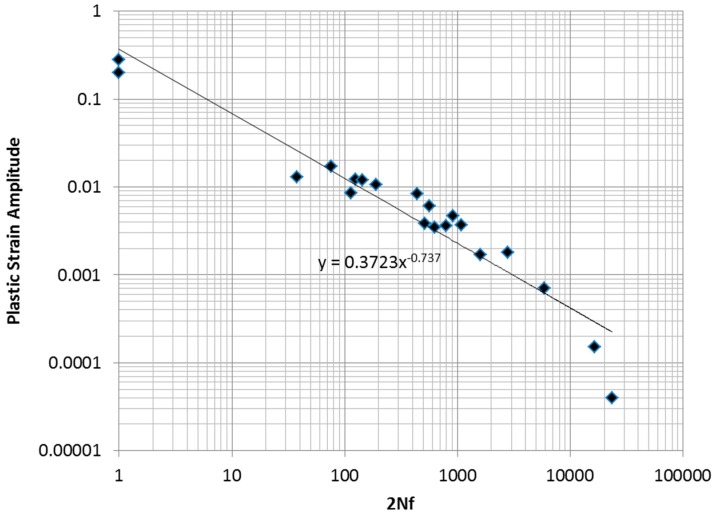
Coffin–Manson plot of data from eighteen low-cycle fatigue tests and two monotonic tests of aluminum 2024-T351 (R^2^ = 0.92). The two data points to the single reversal are from monotonic tests.

In Figure 1, the results from a sequence of low-cycle fatigue tests and two monotonic tension tests on tension specimens of 2024-T351 aluminum are shown. The data is also fitted to a Coffin–Manson model in the figure.

It is clear that the data exhibits a curvature that is not captured by the straight line fit of the Coffin–Manson equation. An ideal model would be one based on a sound physical principle that assures the “best possible” fit to experimentally obtained fatigue test data, considering the statistical uncertainty inherent in the data. An ideal procedure would also provide systematic guidance on constructing the model form. Below, we argue that the maximum entropy concept may provide such a guiding principle.

The concept of entropy occurs in two different contexts in the literature reviewed below. The first case is represented by applications of a class of statistical methods based on information entropy (reviewed in detail in the following section), which may be applied to fatigue data or any other experimental data with inherent uncertainty. These applications may not refer to the physical entropy of the material. Alternatively, the physical entropy at a material point in a device or structure may be used to model the progress of damage at that point. In the latter instance, the process of damage and degradation in material behavior is a fundamental consequence of the second law of thermodynamics, resulting in the increase in entropy of isolated systems with time [3]. In contrast to the more commonly used parameters of stress and plastic strain, the argument is that specimen entropy has a deeper connection to the physics of the damage process.

One of earliest studies to use maximum entropy (or max entropy) probabilistic distributions to study fatigue fracture is [4]. Entropy as a purely statistical concept is used in [5] to model the variability of fatigue crack growth. A version of the maximum entropy method is shown to be a viable alternative to Bayesian updating for analyzing an evolving data population. However, the authors do not connect the concept of entropy to material damage. In [6], the maximum entropy method was used to build a statistical model of the strength distribution in brittle rocks. Since maximum entropy represents a general principle that can lead to many possible probabilistic distributions based on the choice of constraints, studies in the literature have included attempts at specifying constraints on either two or even four moments of the distribution [4,7] in an attempt to compute the parameters of the distribution. In general, in [5,6,7], the thermodynamic entropic dissipation at a material point is not directly used to build a predictive fatigue life relationship.

Basaran and co-workers were among the first to make a connection, using the Boltzmann entropy formula, between physical entropy as measured by plastic dissipation and damage in ductile alloys [8,9]. Later, Khonsari and co-workers [10,11] demonstrated that the thermodynamic entropy generated during a low-cycle fatigue test can serve as a measure of degradation. They proposed that the thermodynamic entropy is a constant when the material reaches its fracture point, independent of geometry, load, and frequency. The hypothesis on critical thermodynamic entropy was tested in [10] on aluminum 6061-T6 through bending, torsion, and tension–compression fatigue tests. In our prior work [12], we used the maximum entropy statistical framework to derive a fatigue life model using material entropy as a predictive variable. This approach is inspired by the work of Jaynes [13], where the information theory concept of entropy was applied to the energy levels of a thermodynamic system, showing that known results from statistical mechanics could be obtained. Information theory entropy was, thus, proportional to thermodynamic entropy. While in some papers [8,9] the accumulated damage is empirically related to entropic dissipation, in [12], the damage D(t) naturally results from the maximum entropy probability distribution as the corresponding cumulative distribution function (CDF). The fatigue life model in [12] is expressed as a damage function and is given in Equation (2) below. The authors describe this approach as a maximum entropy fracture model.

(2) $$D\left(t\right)=1-\exp\left(-\frac{W_{t}}{\rho Tk_{\psi }}\right)$$

**Figure 2:**
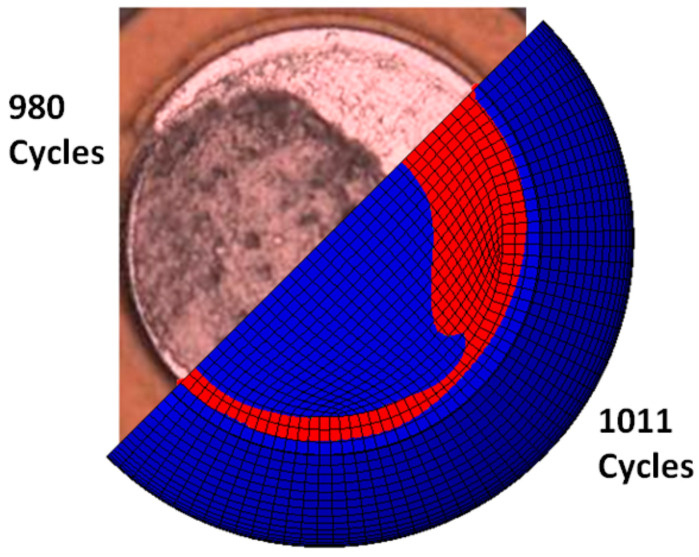
Comparison of crack fronts predicted by a single-parameter maximum entropy model against the experimentally observed creep–fatigue crack in a Sn3.8Ag0.7Cu solder joint under thermal fatigue cycling [12] (reproduced with permission). The single maximum entropy model parameter was extracted using isothermal mechanical tests.

In Equation (2), the damage parameter D(t) is the non-decreasing CDF that ranges from zero (virgin state) to one (failed state). The independent variable is the inelastic dissipation in the material, which is proportional to the entropy of the material through the J2 plasticity theory and the Clausius–Duhem inequality. The single material parameter $k_{\psi }$ in Equation (2) was obtained from isothermal mechanical cycling tests and then used to model fatigue crack propagation under thermal cycling conditions in an electronic assembly. Figure 2 shows a comparison of the estimated and actual number of cycles, as well as crack fronts, at an intermediate stage, with the same area of cracks from both the finite element simulation and thermal cycling fatigue test. To the best of the authors’ knowledge, such a connection between physical entropy dissipation and fatigue crack propagation in ductile alloys has not been made in prior literature.

In [12], it is demonstrated that it is possible to follow the physical process of fatigue crack propagation as a maximum entropy process. However, the arguments that led to the formation of Equation (2) assumed a constant dissipation rate, which in turn implies an exponential distribution for the form of the statistical distribution. More generally, while the use of maximum entropy principles provides the theoretical advantage of being maximally “non-committal” on the data that are unavailable from the experiments [13], the assumption of exponential distribution may be restrictive. Arguably, other distributions that conform to the max entropy principle may provide a better description of damage. However, systematic exploration of such maximum entropy functions, as well as thermodynamic entropy, in describing metal fatigue life data sets appears to be limited in the literature. Thus, in this paper, building on our prior work, we propose the development of a systematic procedure for development of maximum entropy models for describing metal fatigue based on measured thermodyanamic entropy. We demonstrate the approach using low-cycle fatigue experimental data for aluminum 2024-T351 material, and generalize the application of the maximum entropy principle using a broader class of statistical distributions, including the truncated exponential and the truncated normal distribution. We begin first with a brief review of the maximum entropy principle.

## 2. A Review of the Maximum Entropy Principle 

The concept of entropy as applied to heat engines is due to Clausius, but the connection of entropy to the probability of the states of a thermodynamic system began with Boltzmann. Boltzmann demonstrated that the second law of thermodynamics for an ideal gas is a consequence of the mechanics of the collisions of the molecules [14]. He showed that a sufficiently large number of interrelated deterministic events will result in random states. He derived the following function, given in Equation (3), for a uniform distribution, and argued that this quantity had the same physical meaning as the entropy proposed by Clausius. This led to the Boltzmann H function:

(3) $$H\left(p\right)=\underset{i}{\sum }p_{i}\ln p_{i}\;\;p_{i}=p=\frac{1}{n}$$

The above expression is closely related to Gibb’s entropy formula:

(4) $$S\left(p\right)=-k_{b}\underset{i}{\sum }p_{i}\ln p_{i}$$

Shannon’s research in information theory led to a mathematical expression (discussed later in Equation (6)) that is strikingly similar to the thermodynamic entropy formulas of Boltzmann and Gibbs, described above. It is important to note that Shannon’s argument was a purely statistical one and no physical significance was claimed. It was not until the work of Jaynes [13] that a connection between the information entropy of Shannon and thermodynamic entropy was established.

Here, we describe the abstract development of Shannon’s formula based on a counting argument [15], considering the information content of a whole number, which can range in value from 0 to N. If we claim that each digit of the number is a unit of information, then it clearly takes $\log_{b}N$ digits to represent the number in a base b system. If the base of the logarithm is changed, the resulting information will change by a constant, but the ratios of information for different N will be preserved, provided the same base is used for all of them. Thus, $\log_{b}N$ is a reasonable measure of the information contained in a variable, which can range from 0 to N. If we consider a random experiment with N possible equally likely, mutually exclusive outcomes, then the information contained in a given outcome is still $\log_{b}N=-\log_{b}p$, with p being the probability of the event. We argue that the information in a given event is strictly determined by p, regardless of how the remaining 1−p probability is allocated to other events. Thus, even if the events do not have equal probabilities, the information for any given event is still $-\log_{b}p$ [15]. This function has the expected property that the information contained in the occurrence of two (or more) statistically independent events is the sum of the information in each of the events separately, as shown below in Equation (5). This property is fundamentally important (as pointed out in [13]) and further reinforces the argument for the $-\log_{b}p$ measure of information.

(5) $$\begin{array}{c} I\left(p\right)=-\ln p_{i}\\ I\left(p_{i}p_{j}\right)=I\left(p_{i}\right)+I\left(p_{j}\right)\;\;\;\;\;:i\neq j \end{array}$$

If the events correspond to a discrete random variable, then they must be mutually exclusive, and the probability of the union of the sequence of the events is equal to one [16]. The entropy of the density function is taken as the expected value of the information in the events [17]. This leads to the Shannon information entropy formula:

(6) $$H\left(p\right)=E\left[I\left(p\right)\right]=-\underset{i}{\sum }p_{i}\ln p_{i}$$

This function (and only this function) satisfies these three conditions:

1. Continuity: It is a continuous function of the $p_{i}$;
2. Monotonicity: It is an increasing function of *n*, if all the $p_{i}$ are equal;
3. Composition: If an event can be decomposed into two or more lower level events, the function H(p) will evaluate this identically, whether the lower or higher level events are used in the computation, provided that the appropriate conditional probabilities are used to relate the higher and lower level events.

Jaynes [13] noted that there is a symbolic similarity between the expressions for thermodynamic (Gibbs) entropy (Equation (3)) and Shannon’s information entropy (Equation (6)), but commented that the similarity did not necessarily imply a deeper connection. Jaynes then proceeded to show that a connection did exist and that many results of statistical thermodynamics could be interpreted as applications of Shannon’s information entropy concept to physical systems. The expression for the Gibbs entropy is the result of a development involving various physical assumptions—some based on experimental evidence, and some not. Conversely, Shannon’s entropy is based on mathematical and logical reasoning, not physical evidence. Shannon’s model was developed to model the abstract mathematical properties of digital communication, and prior to Jaynes, was not claimed to be applicable to the physical sciences. Shannon defined the entropy of a discrete probability distribution as Equation (6).

The maximum entropy method as set forth by Jaynes is as follows [13]: The probability mass function that maximizes Equation (6), subject to constraint from Equations (7) and (8), is the best choice if no other information is available to specify the probability distribution.

(7) $$\underset{i}{\sum }p_{i}=1$$

(8) $$E\left[g\left(x_{i}\right)\right]=\underset{i}{\sum }p_{i}g\left(x_{i}\right)\;\;\;\;\;:x_{i}\in \left\{x_{1},x_{2},\ldots x_{i}\ldots x_{m}\right\}$$

where $E\left[g\left(x_{i}\right)\right]$ is the expected value of, $g\left(x_{i}\right)$. The following probability mass function (Equation (9)) can be shown to maximize Equation (6):

(9) $$p_{i}=e^{-\lambda_{0}-\lambda_{1}g\left(x_{i}\right)}$$

The constants $\lambda_{0}$ and $\lambda_{1}$ are Lagrange multipliers associated with the constraints. Jaynes calls this approach the maximum entropy method and calls the derived probability functions maximum entropy distributions (MaxEnt method and MaxEnt distributions). Multiple expected value constraints may be applied (not simply moments, as is common in probability analysis), resulting in the following form of the MaxEnt distribution:

(10) $$p_{i}=e^{-\lambda_{0}-\lambda_{1}g_{1}\left(x_{i}\right)-\ldots -\lambda_{m}g_{m}\left(x_{i}\right)}$$

The entropy of the resulting distribution is [13]:

(11) $$S_{max}=\lambda_{0}+\lambda_{1}E[g_{1}\left(x\right)]+\ldots +\lambda_{m}E[g_{m}\left(x\right)]$$

Jaynes’s argument was for the discrete case. The entropy of a continuous probability density function is also known and is defined as [16]:

(12) $$H\left(f\left(x\right)\right)=-\int_{-\infty }^{\infty }f\left(x\right)\ln f\left(x\right)dx$$

The corresponding continuous version of Equation (10) is given below [16]:

(13) $$f\left(x\right)=e^{-\lambda_{0}-\lambda_{1}g_{1}\left(x\right)-\ldots -\lambda_{m}g_{m}\left(x\right)}$$

One important point regarding Equation (13) is that it is only a probability density function for specific values of the parameters $\lambda_{k}$. This situation differs from the usual approach to representing probability density functions or distribution functions, where the functions are admissible for ranges of parameter values. Additionally, the method Jaynes sets forth assumes that the values used for moment function constraints are not estimates subject to sampling variation. They are taken as essentially exact values of the distribution moment functions. This assumption differs from traditional inferential statistics, where moments or quantiles are estimated from data and sampling errors are estimated.

Jaynes showed that if we choose the probability distribution for the system microstates based on maximizing Shannon entropy, known results from statistical mechanics can be obtained, without new physical assumptions, and in particular, the thermodynamic entropy of the system is found to be the Gibbs entropy of Equation (4). Shannon’s entropy for the distribution is proportional to the physical entropy of the system, however, only if the probability distribution is applied to the thermodynamic states of the system. Jaynes [13] argues that this shows that thermodynamic entropy is an application of a more general principle. Further to this point, Jaynes argues that if a probability model is required for some application, where certain expected values are known but other details are not, the maximum entropy approach should be used to find the probability distribution. Jaynes uses the term “maximally non-committal” to describe probability distributions obtained by this process. What is known about the random variable in question is captured in mathematical constraints, while the principle of maximum entropy accounts for what is not known. While information entropy is only proportional to thermodynamic entropy in certain circumstances, Jaynes argues that choosing the probability density function that maximizes the Shannon entropy subject to various constraints is appropriate to any situation where a reference probability distribution is needed. The application could be physical or not, and need not necessarily have a relationship to thermodynamic states.

## 3. Maximum Entropy Distributions

We argue that if a given parametric family of distributions is selected for some reason (as is common practice), then within that family of distributions we should prefer the parameter values that maximize entropy (subject to any constraints) over those that do not. For example, if the Weibull distribution has already been chosen for some application, and the characteristic life is known, then the Weibull exponent should be chosen to maximize entropy. It is noteworthy that the exponential distribution and the normal distribution are the MaxEnt distributions corresponding to a prescribed mean value and to the prescribed mean and variance values, respectively [18]. Given the fundamental importance of these distributions in statistical theory, it is informative that they can be directly derived from the principles of maximum entropy. Just as Jaynes showed that statistical thermodynamic results derivable by other means could be obtained from maximum entropy methods, it has also been shown that the well-known and fundamental normal distribution, traditionally derived by other means, can also be based on a maximum entropy argument. Even the Weibull distribution can be derived from a maximum entropy approach if appropriate moment functions are chosen [18]. These MaxEnt distributions are listed in Table 1.

Note the references to truncated distributions in Table 1. A distribution is described as truncated if the value of its density or mass function is forced to zero (when otherwise it would be non-zero) outside of a specific range. Thus, the truncated normal distribution functions can be thought of as ordinary normal probability density functions (PDFs) that are clipped to zero probability outside of their non-zero range. As described later, they are multiplied by a normalizing constant to correct for the missing density. Truncation at x=0 is necessary for applications to non-negative variables. The cumulative distribution function (CDF) of a truncated normal random variable has a finite slope at x=0. If a second truncation at x=a is specified, then the CDF is forced to be exactly equal to 1 for all x≥a. We begin the discussion of MaxEnt distributions with the truncated exponential distribution.

### 3.1. MaxEnt Form of Truncated Exponential Distribution

**Figure 3:**
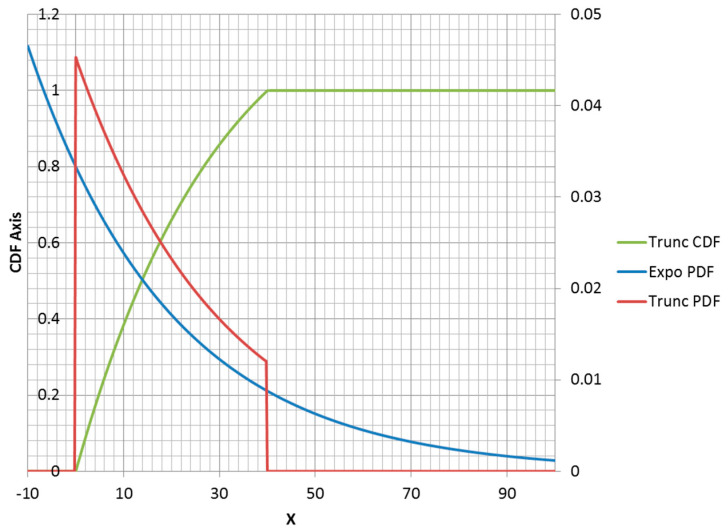
Truncated exponential distribution with *λ* = 0.03; *a* = 40. Note: CDF = cumulative distribution function; PDF = probability density function; trunc = truncated; expo = exponential.

**Figure 4:**
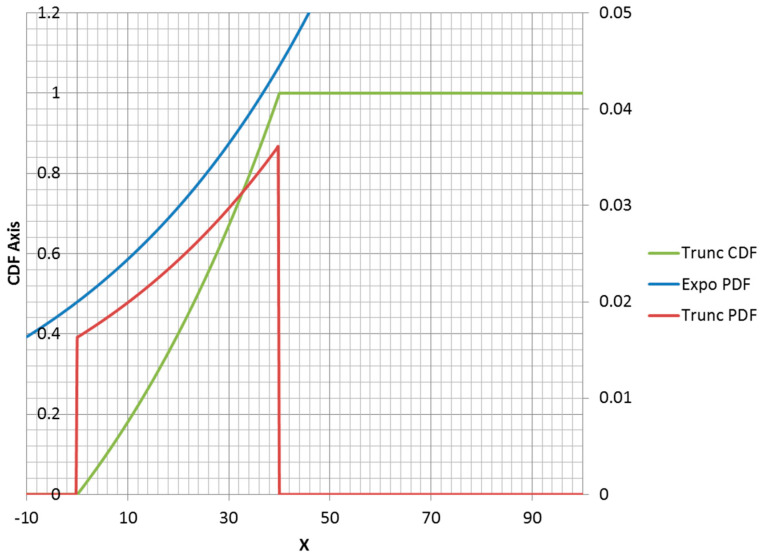
Rising truncated exponential distribution with *λ* = −0.02; *a* = 40.

The truncated exponential distribution can be constructed in an analogous fashion for positive values of λ (parent PDF is a decreasing function). An example is plotted in Figure 3. However, it is possible for a truncated exponential distribution to be an increasing function within its non-zero range (Figure 4). Clipping the positive exponent at some specified value enables its use as a PDF. This corresponds to a negative-valued lambda, which is not admissible in the non-truncated case. If the specified mean was to the right of the midpoint of the non-zero range, then the lambda would be negative.

It should also be noted that changing the location of a distribution function without changing its shape has no effect on the entropy value. Thus, a left endpoint other than zero could be used for any of the distributions that have zero value for negative x. Naturally, this shift would change the moment function values. Note that specifying a right truncation value changes the shape of the remaining distribution function and should be thought of as adding an extra parameter. Thus, a truncated exponential distribution is a two-parameter distribution.

Below is the truncated exponential distribution for PDF and CDF:

(14) $$\begin{array}{ccc} f_{trunc}\left(x,\lambda ,a\right)=\frac{\lambda \;Exp\left(-\lambda x\right)}{1-Exp\left(-\lambda a\right)} & for & 0\leq x\leq a\\ F_{trunc}\left(x,\lambda ,a\right)=\frac{1-Exp\left(-\lambda x\right)}{1-Exp\left(-\lambda a\right)} & for & 0\leq x\leq a \end{array}$$

Below is the expected value of a truncated exponential random variable:

(15) $$E\left(x\right)=\frac{1}{\lambda }\left(\frac{1-\left(\lambda a+1\right)Exp\left(-\lambda a\right)}{1-Exp\left(-\lambda a\right)}\right)$$

**Figure 5:**
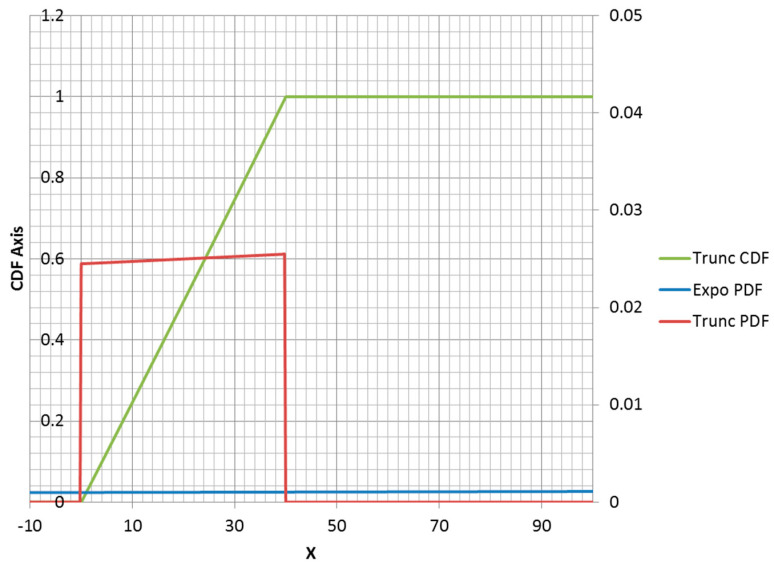
Rising truncated exponential distribution with *λ* = −0.001; *a* = 40.

Note that the uniform distribution is a limiting case of the truncated exponential distribution and corresponds to the lambda approaching zero. An example is shown in Figure 5.

(16) $$\begin{array}{c} \underset{\lambda \to 0}{\lim}f_{trunc}\left(x,\lambda ,a\right)=\frac{1}{a}\;\;\;\;for\;\;0\leq x\leq a\\ \underset{\lambda \to 0}{\lim}E\left(x\right)=\frac{a}{2} \end{array}$$

### 3.2. MaxEnt Form of Truncated Normal Distribution

**Figure 6:**
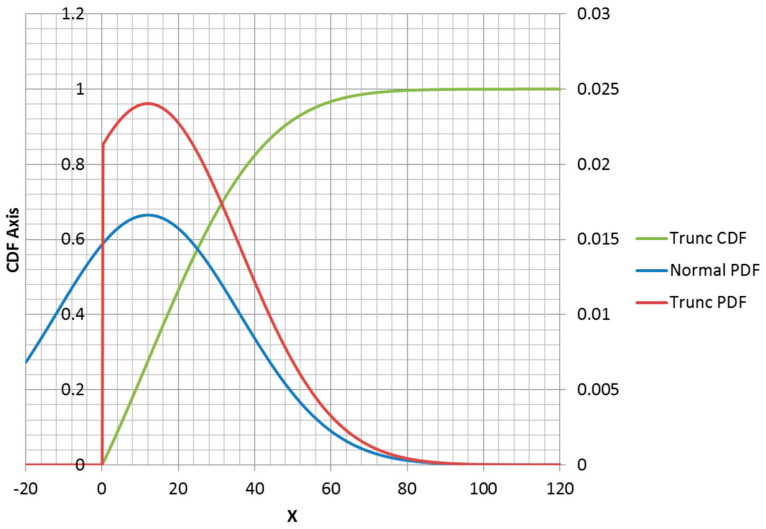
Truncated normal distribution plotted with the parent (non-truncated) normal distribution. Density correction for x≥0 is equal to 1.23.

**Figure 7:**
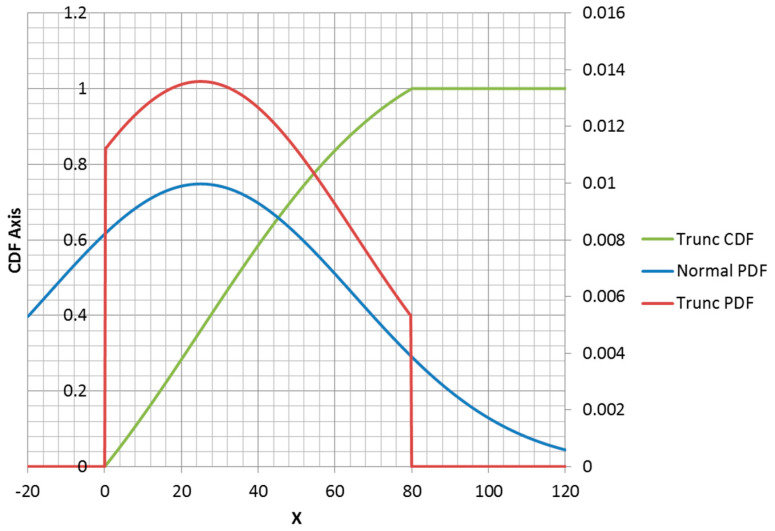
Left and right truncated normal distribution.

The truncated normal distribution can be explained in terms of the normal PDF. For x≥0, the PDF has the same shape as a non-truncated normal PDF, but scaled to make up the density lost for x<0 (Figure 6). The truncation of the portion of the density less than zero changes the mean and standard deviation from the parameters that the truncated distribution inherits from the normal distribution. Adding a second truncation point at x=a forces the function to be equal to 1 for all x≥a and adds a corner to the CDF at x=a (Figure 7). Additionally, the correction factor must be larger to correct for missing density x<0 and also x≥a.

The PDF and the CDF for the left truncated normal distribution can be shown to be:

(17) $$\begin{array}{c} Normal\;CDF\;in\;terms\;of\;standard\;normal\;CDF\\ F_{norm}\left(x,\mu ,\sigma \right)=\Phi \left(\frac{x-\mu }{\sigma }\right)\\ F_{trunc}\left(x,\mu ,\sigma \right)=\frac{F_{norm}\left(x,\mu ,\sigma \right)-F_{norm}\left(0,\mu ,\sigma \right)}{1-F_{norm}\left(0,\mu ,\sigma \right)}\;\;for\;x\geq 0\\ f_{trunc}\left(x,\mu ,\sigma \right)=\left(\frac{1}{1-F_{norm}\left(0,\mu ,\sigma \right)}\right)\frac{1}{\sigma \sqrt{2\pi }}Exp\left(-\frac{{\left(x-\mu \right)}^{2}}{2\sigma^{2}}\right)\;\;for\;x\geq 0 \end{array}$$

The factor in the denominator of the CDF definition in Equation (17) is the area correction factor C.

(18) $$C=\frac{1}{1-F_{norm}\left(0,\mu ,\sigma \right)}$$

Truncated normal distribution in two-parameter MaxEnt form is:

(19) $$\begin{array}{c} f_{trunc}\left(x,\mu ,\sigma \right)=Exp\left(-\lambda_{0}-\lambda_{1}x+\lambda_{2}x^{2}\right)\\ \lambda_{0}=-\frac{\mu^{2}}{2\sigma^{2}}-\ln\left(\frac{C}{\sigma \sqrt{2\pi }}\right)\;\;\;\;\lambda_{1}=-\frac{\mu }{\sigma^{2}}\;\;\;\;\lambda_{2}=\frac{1}{2\sigma^{2}} \end{array}$$

Thus, just as the normal distribution is MaxEnt for moment functions *x*, *x*^2^, where *x* ranges over (−∞, ∞), the truncated Normal distribution is MaxEnt for the same moment functions over the range [0, ∞). Note that the μ and σ are the mean and standard deviation of the parent (un-truncated) normal distribution, not the truncated normal distribution.

### 3.3. MaxEnt Form of the Weibull Distribution

Since the Weibull distribution is widely used, it is useful to know what parameter value choices maximize the entropy of the function. It is often the case that only one of the two parameters is known and we seek a rational approach to assigning a value to the second parameter. In this case, we suggest that choosing the parameter value that maximizes the entropy of the distribution is the correct approach.

**Figure 8:**
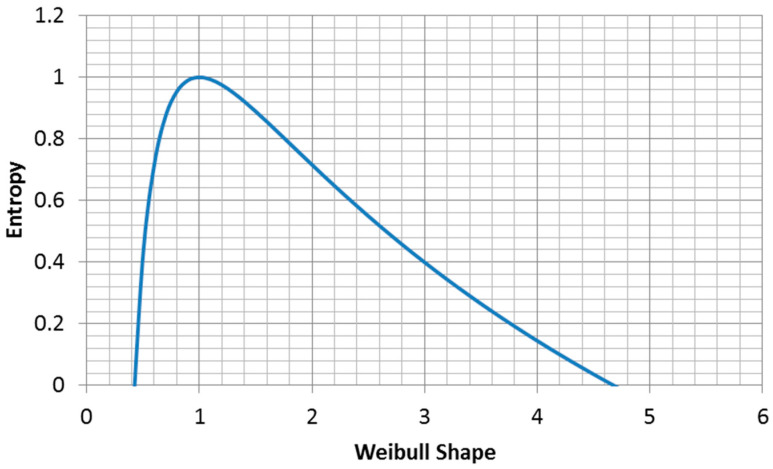
Entropy of a Weibull distribution with a fixed mean.

The entropy of the Weibull distribution is (Figure 8, derived from Equation (2.80c) in [21]):

(20) $$\begin{array}{c} H=\gamma \left(1-\frac{1}{\alpha }\right)+\ln\left(\frac{\beta }{\alpha }\right)+1\\ \gamma =0.577216\ldots \;\;Euler^{\prime }s\;constant \end{array}$$

The mean of a Weibull distribution is [21]:

(21) $$\mu =\beta \Gamma \left(1+\frac{1}{\alpha }\right)$$

Thus, the entropy for a Weibull distribution with a fixed mean (moment constraint on *x*) is:

(22) $$H_{\mu }=\gamma \left(1-\frac{1}{\alpha }\right)+\ln\left(\mu \right)-\ln\left(\Gamma \left(1+\frac{1}{\alpha }\right)\right)-\ln\left(\alpha \right)+1$$

Here, we maximize the entropy function:

(23) $$\frac{dH_{\mu }}{d\alpha }=0$$

Then, we recall the properties of the digamma function [21]:

(24) $$\begin{array}{c} \psi \left(x\right)=\frac{d}{dx}\left[\ln\left(\Gamma \left(x\right)\right)\right]\\ \psi \left(1+x\right)=\psi \left(x\right)+\frac{1}{x} \end{array}$$

Therefore:

(25) $$\begin{array}{c} \frac{dH_{\mu }}{d\alpha }=\frac{\gamma }{\alpha^{2}}+\frac{\psi \left(1+\frac{1}{\alpha }\right)}{\alpha^{2}}-\frac{1}{\alpha }=0\\ \psi \left(\frac{1}{\alpha }\right)=-\gamma \end{array}$$

This is only true for α=1. Thus, within the Weibull family of distributions, for a given fixed mean, the exponential distribution has the highest entropy, in agreement with Jaynes’s result.

**Figure 9:**
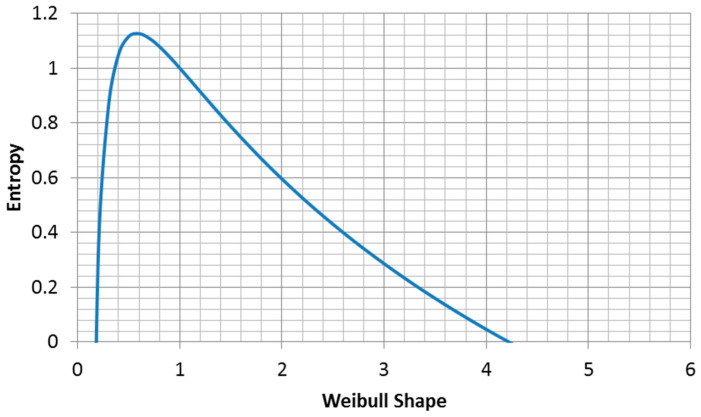
Plot of Equation (26) for β=1.

The maximum entropy for fixed characteristic life is (Figure 9):

(26) $$H=\gamma \left(1-\frac{1}{\alpha }\right)+\ln\left(\frac{\beta }{\alpha }\right)+1\;\;for\;\beta =const.$$

Proceeding as above:

(27) $$\begin{array}{c} \frac{dH}{d\alpha }=\frac{\gamma }{\alpha^{2}}-\frac{1}{\alpha }=0\\ \gamma =\alpha \end{array}$$

Thus, for the fixed characteristic life case, α=γ (the Euler’s constant).

## 4. Application of Maximum Entropy to Low-Cycle Fatigue of 2024-T351 Aluminum

**Figure 10:**
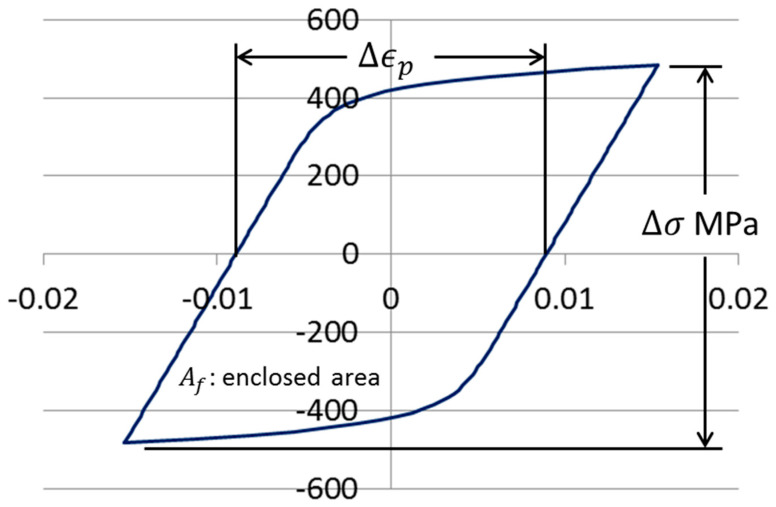
Stress–strain loop showing plastic strain. The variables are defined in Table 2.

When a specimen is subjected to axial load cycles of a magnitude sufficient to cause plastic deformation, the stress–strain history for the specimen can frequently be described as a loop, as shown in Figure 10. To determine the fatigue life of the specimen, the load cycles are applied until the specimen fails, or until its compliance exceeds some proportion of its initial compliance. The Coffin–Manson relationship (Equation (1)) is commonly used to model the relationship between plastic strain range and reversals to failure. The parameter $\epsilon_{f}\prime$ is determined by fitting the curve to fatigue data. It is frequently close in value to $\epsilon_{f}$.

As mentioned earlier, a sequence of low-cycle fatigue tests, along with two monotonic tension tests, was performed on tension specimens of 2024-T351 aluminum. Eighteen specimens were tested under constant-amplitude, fully reversed fatigue conditions. In five cases, representative stress–strain loops were collected at various cycle intervals. Two specimens were tested to failure monotonically. The data collected is summarized in Table 2. The data is fitted to a Coffin–Manson model, as shown in Figure 1.

As mentioned earlier, the data exhibits a curvature that is not captured by the straight line fit of the Coffin–Manson power law. An alternative approach to modeling data such as this, using concepts developed from maximum entropy, is developed below. The authors of [12] showed that material entropy is proportional to inelastic dissipation in experiments such as this, where the temperature of the specimens is essentially constant. Thus, inelastic dissipation is exploited as a surrogate for entropy in the development that follows.

The variable D representing the ability of the material at a point to bear load is fundamental in the literature of damage mechanics [23]. The value of D=0 (undamaged) represents virgin material, while D=1 is taken to correspond to failed material. The variable D is a non-decreasing quantity, since damage is inherently irreversible. The Coffin–Manson equation can be rewritten in terms of damage, and doing so will be shown to provide a departure point for further development. We begin by rearranging Equation (1) into the following form:

(28) $$\frac{1}{2N_{f}}={\left(\frac{\Delta \epsilon_{p}}{2\epsilon_{f}^{\prime }}\right)}^{-\frac{1}{c}}$$

Depending on the application, the damage variable D may be expressed as a function of various independent variables. In fatigue applications, it is common to use the following (applicable to constant damage per load cycle) Palmgren–Miner definition of damage. It is understood that $N_{f}$ may depend on other variables, such as temperature or plastic strain amplitude.

(29) $$D\left(N\right)=\frac{N}{N_{f}}$$

We can write the damage accumulation per reversal:

(30) $$D_{rev}=\frac{1}{2N_{f}}$$

Finally, Equation (28) can be recast as a damage equation as follows:

(31) $$D_{rev}={\left(\frac{\Delta \epsilon_{p}}{2\epsilon_{f}^{\prime }}\right)}^{-\frac{1}{c}}=f\left(\Delta \epsilon_{p}\right)$$

where f(·) denotes a functional relationship with the argument. Following [12], we propose developing a function of the form of Equation (31), in terms of energy per reversal rather than plastic strain range. This relationship will have the form:

(32) $$D_{rev}=f\left(\frac{W_{f}}{2N_{f}}\right)$$

In the development that follows, a general approach to deriving functions of the form of the above equation will be proposed. In order to apply an equation of the above form to the data in Table 3, we first need to determine the inelastic dissipation per reversal corresponding to each of the test conditions of the form shown in Figure 10. The energy expended in inelastic dissipation for a cyclic test under constant conditions is given by the area enclosed by the loop. Note that in Table 3, actual loop data was only available for five of the 20 tests. In all cases, the plastic strain range and stress range (and reversals to failure) were collected. Fortunately, the shapes of the loops follow known trends, and thus it was possible to deduce the inelastic dissipation for the tests where loops were not available for measurement. The inelastic dissipation for the two monotonic tests was also deduced from the available loop data, although a different analytical approach was used.

**Figure 11:**
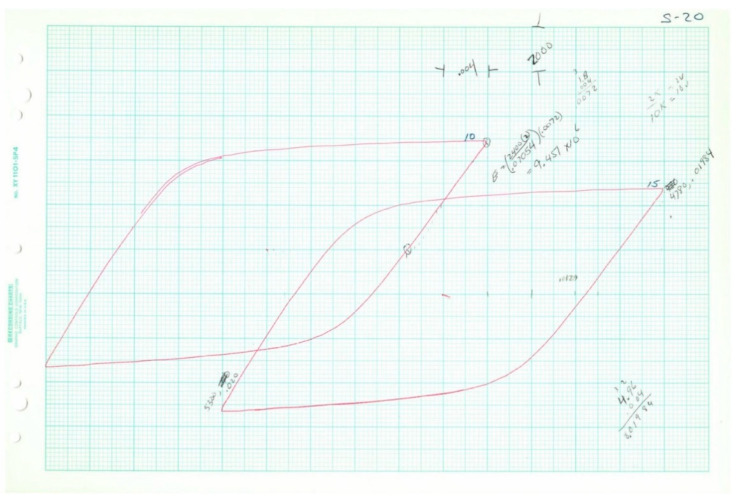
Raw data plotted on chart paper from Test 4, 2*N_f_* = 38.

**Figure 12:**
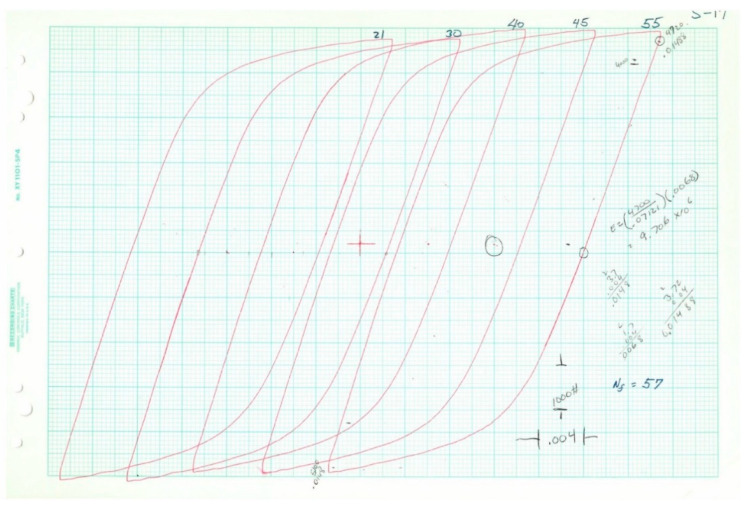
Raw data plotted on chart paper from Test 8, 2*N_f_* = 114.

**Figure 13:**
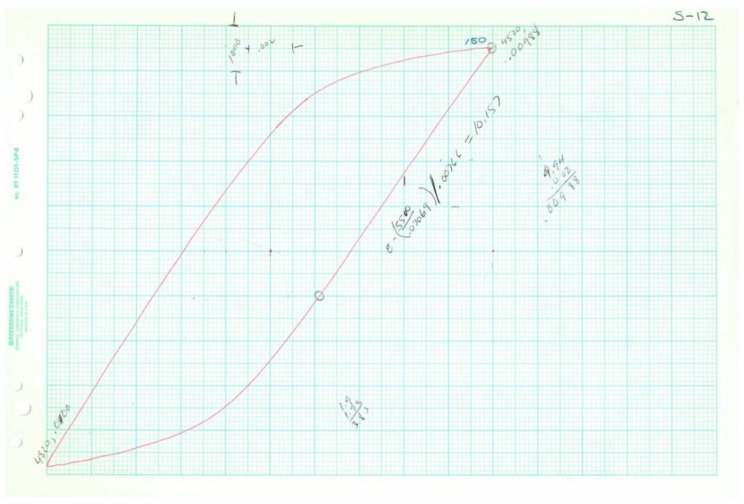
Raw data plotted on chart paper from Test 12, 2*N_f_* = 516.

**Figure 14:**
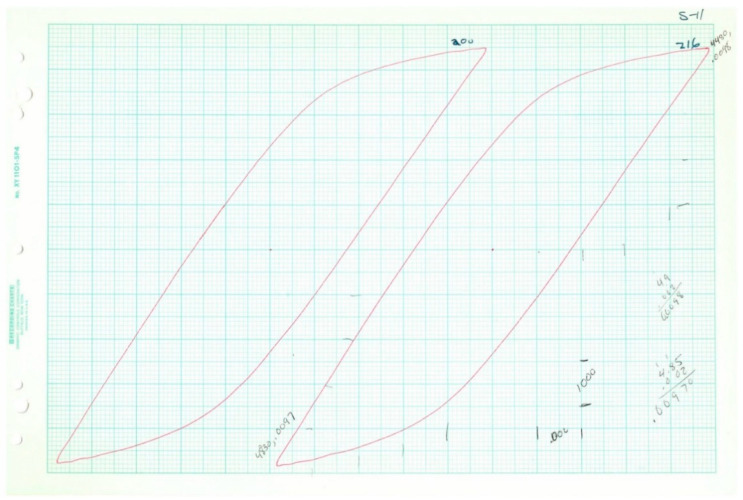
Raw data plotted on chart paper from Test 15, 2*N_f_* = 624.

**Figure 15:**
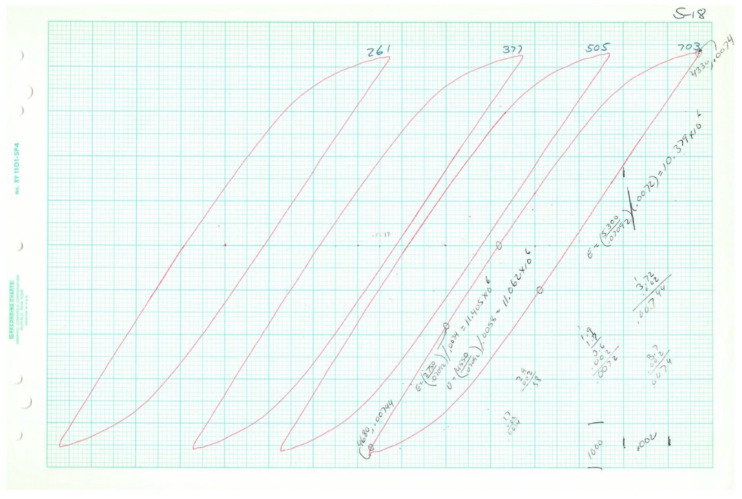
Raw data plotted on chart paper from Test 17, 2*N_f_* = 1608.

Plotted loops for the five loop data samples are given below in Figure 11, Figure 12, Figure 13, Figure 14 and Figure 15. In each case, several loops were provided.

The Ramberg–Osgood relationship (Equation (33)) is frequently successful for modeling data such as this. This model assumes that the plastic portion of the strain range is a power law of the stress range. There is no explicit yield point with this model. The total strain range is given by Equation (34) and is used to model the shapes of the loops. For the purposes of fitting Equation (34), the origin of the stress and strain range variables is placed at the lower left corner of the loop.

The Ramberg–Osgood plasticity model for stress–strain loops is [23]:

(33) $$\Delta \epsilon_{p}={\left(\frac{\Delta \sigma }{K}\right)}^{\frac{1}{n}}$$

(34) $$\Delta \epsilon_{total}=\frac{\Delta \sigma }{E}+{\left(\frac{\Delta \sigma }{K}\right)}^{\frac{1}{n}}$$

**Figure 16:**
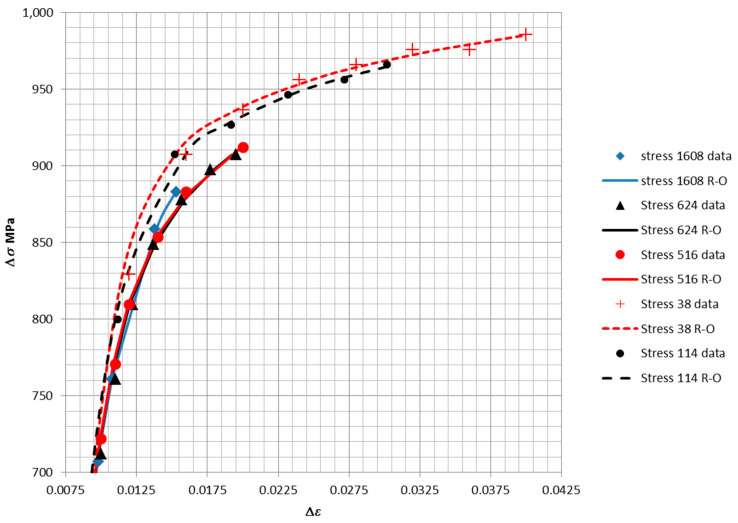
Fit of Equation (34) to 5 data sets (*E* = 73.800 GPa for all fits) (R^2^ = 0.997).

The fits of Equation (34) to loop data were performed using the least squares approach and is shown in Figure 16. The fits to the data were of high accuracy, as demonstrated by the R^2^ value of 0.997. This confirms that Equation (34) provides a reasonable model of the shape of the loops in Figure 11, Figure 12, Figure 13, Figure 14 and Figure 15. The points are samples measured from the loops, while the line is the fit of Equation (34). A separate fit was performed for the parameters in Equation (34) for each of the five loops. A common value of Young’s modulus was fit simultaneously to the five sets of data. Specific values of *n* and *K* were obtained for each loop.

**Figure 17:**
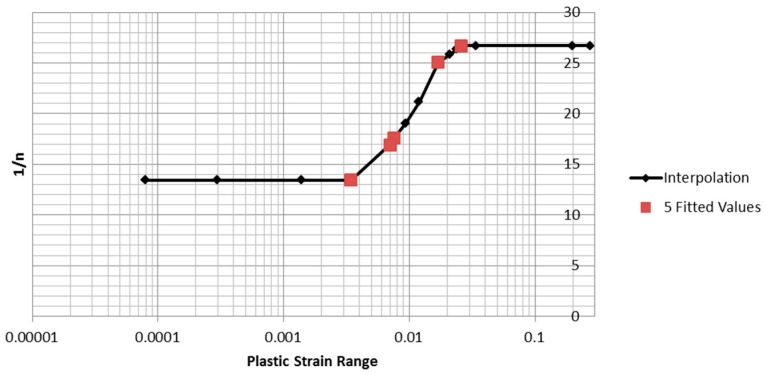
5 Fitted values of *1/n* with interpolation function.

The five sets of parameters obtained from the fitted loops were used to estimate the parameter *1/n* for the remaining 15 tests. The fitted *1/n* value was found to be a strictly increasing function of plastic strain range, and is plotted in Figure 17. The “interpolation” line markers show the values of *1/n* used for the remaining 15 tests. The values were linearly interpolated between the maximum and minimum values. For plastic strain ranges outside the range of the measured data, the value of the nearest measured data value was used. As will be shown below, the predicted inelastic dissipation is mainly determined by the plastic strain range and the stress range, and is only weakly dependent on the value of *1/n* used.

**Figure 18:**
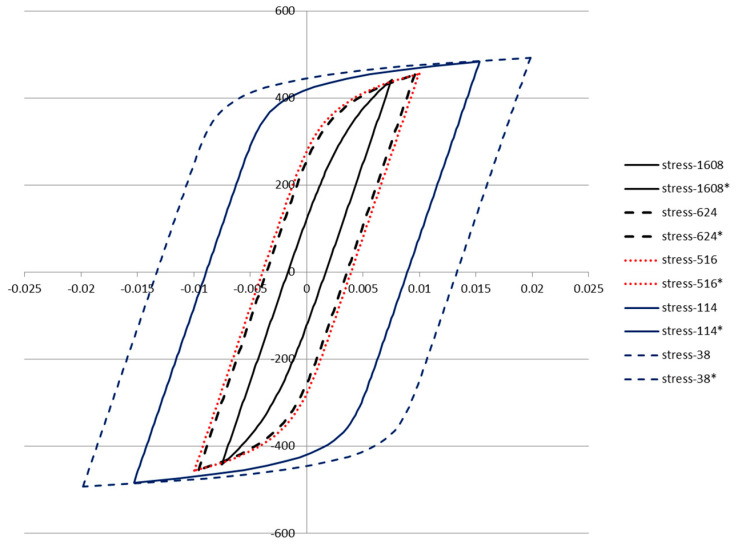
Ramberg–Osgood curves based on loop fits.

The five loops (represented by Equation (34)) are plotted in Figure 18 below using the parameters fit to the corresponding loop data. The inelastic dissipation per cycle is the area enclosed by the loop.

The area of the loop in terms of the parameters in Equation (34) and the loading parameters are given in Equation (35). The form of this equation has the advantage that it is relatively robust to errors in fitting the parameter *n*, since both of the actual measured values of the stress range and strain range are used.

The loop area (dissipation per cycle) in terms of *n* is [23]:

(35) $$A_{f}=\frac{1-n}{1+n}\Delta \sigma \Delta \epsilon_{p}$$

In the present case, we wish to describe the evolution of damage in terms of reversals rather than cycles. It is apparent from Equation (36) that the inelastic dissipation per reversal is half the area of the loop given by Equation (35), and is given in Equation (37).

Total inelastic dissipation in terms of cycles and reversals:

(36) $$W_{f}=N_{f}A_{f}=\left(2N_{f}\right)\left(\frac{1}{2}A_{f}\right)$$

Inelastic dissipation per reversal:

(37) $$\frac{W_{f}}{2N_{f}}=\frac{1-n}{2\left(1+n\right)}\Delta \sigma \Delta \epsilon_{p}$$

For specimens subjected to a monotonic test, the inelastic dissipation is the area under the plastic portion of the stress–strain curve. If the plastic portion of the curve is modeled by an equation of the form of Equation (34), the area under the plastic portion is given by Equation (38). A monotonic test to fracture can be interpreted as a fatigue test, with failure occurring after a single reversal. Thus, the inelastic dissipation per reversal is given by Equation (39):

The monotonic area (dissipation per reversal) in terms of *n*:

(38) $$A_{f}=\frac{1}{1+n}\sigma_{f}\epsilon_{f}$$

The inelastic dissipation for a monotonic test:

(39) $$\frac{W_{f}}{2N_{f}}=A_{f}=\frac{1}{1+n}\sigma_{f}\epsilon_{f}\;\;\;\;2N_{f}=1$$

**Figure 19:**
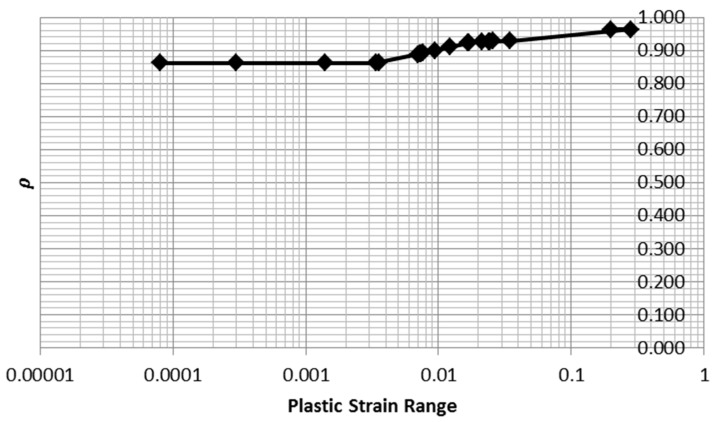
Here, *ρ* is shown as a function of plastic strain range.

Note that in Equations (37) and (39), the area is computed from plastic strain range multiplied by stress range times a factor dependent on *n*. The functions are given in Equation (40) and the values of *ρ* are summarized in Table 4 and plotted in Figure 19.

(40) $$\begin{array}{c} \rho_{mono}=\frac{1}{1+n}\\ \rho_{loop}=\frac{1-n}{1+n} \end{array}$$

Note that the value of *ρ* does not change greatly as *n* is varied. This observation indicates that the computation of areas for the monotonic and cyclic tests is robust to errors in fitting the Ramberg–Osgood parameter *n*. Thus, the inference of inelastic dissipation for the 15 tests for which loop data was not available is justified.

**Figure 20:**
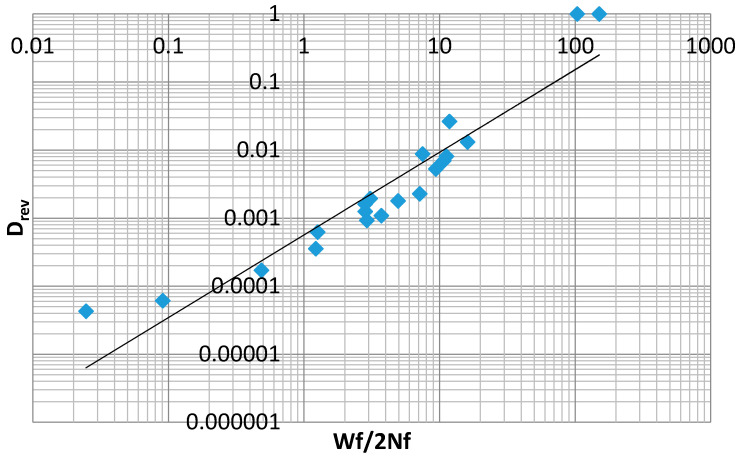
Damage per reversal as a function of inelastic dissipation per reversal with power law fit (R^2^ = 0.89).

Table 3 below includes values computed from Equations (37) and (39) for inelastic dissipation per reversal, as well as damage per reversal, according to Equation (30). These data are plotted in Figure 20. These points represent data corresponding to a relationship with the form of Equation (32). The lack of fit provided by the power law indicates that a different modeling equation is required for data of this type. In the development that follows, various expressions, including some based on MaxEnt principals, will be proposed to model the data plotted in Figure 20.

### Discussion of Candidate Distribution Functions

**Figure 21:**
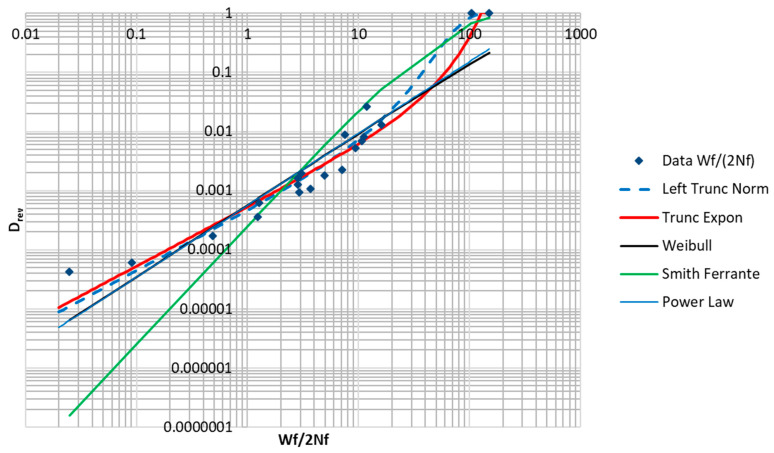
Plots of functions in Table 4.

Inelastic dissipation is a non-negative-valued function, so only distribution functions equal to zero for x≥0 are admissible candidates. Table 4 contains a summary of the fitted functions, as well as the sum of squares of error remaining after the fitting. The natural logs of the data were fitted to the natural logs of the predicted values. Plots of the fitted curves and the data are shown in Figure 21. Only the truncated forms of the normal distribution are considered. Distributions that are truncated on the right, such as the truncated exponential distribution, have the additional advantage that they are strictly equal one for x≥a.

The data set being fitted has some noteworthy features. Even though the data is of low-cycle fatigue, most of the samples still represent very small values of $D_{rev}$. Additionally, the data points show a concave upwards trend that limits the quality of the fit achievable by a power law relationship. The fit was notably better for the right truncated exponential distribution with a negative λ. The fitting procedure converged to a negative λ, which corresponds to a rising exponential curve that becomes constant at $D_{cycle}=1$. The best fits were achieved by the truncated normal distribution and the truncated exponential distribution. The Smith–Ferrante function (popular in cohesive zone models of fracture) is typically used to represent the traction versus separation, and is founded on the relationship binding materials together at the microscopic scale [24]. Its integral is used here, which has the qualitative features of a damage function. The Weibull distribution function was also tried. Additionally, a power law expression having the form of the Coffin–Manson relation was tried. This function would be truncated at $D_{rev}=1$.

Note that the Coffin–Manson expression typically relates plastic strain range to cycles to failure. In Table 4, it is shown in an inverted form and expressed in terms of $W_{c}$. It is clear from the sum of squared error column in Table 4 and from Figure 21 below that the truncated normal distribution provided the best fit to the data, followed by the truncated exponential distribution. The (inverted) Coffin–Manson expression and the Weibull distribution function provided the next best fits.

Parameters fit by numerical solver to the fatigue data for the truncated normal distribution (Equation (41)) and the truncated exponential distribution (Equation (42)) are given below:

(41) $$D_{rev}=\frac{\;F_{norm}\left(x,72.1,\;27.3\right)-F_{norm}\left(0,72.1,\;27.3\right)}{1-F_{norm}\left(0,72.1,\;27.3\right)}\;\;for\;x\geq 0$$

(42) $$D_{rev}=\frac{1-Exp\left(0.0325x\right)}{1-Exp\left(\left(-0.0325\right)\left(127.2\right)\right)}\;\;\;\;for\;\;0\leq x\leq 127.2$$

**Figure 22:**
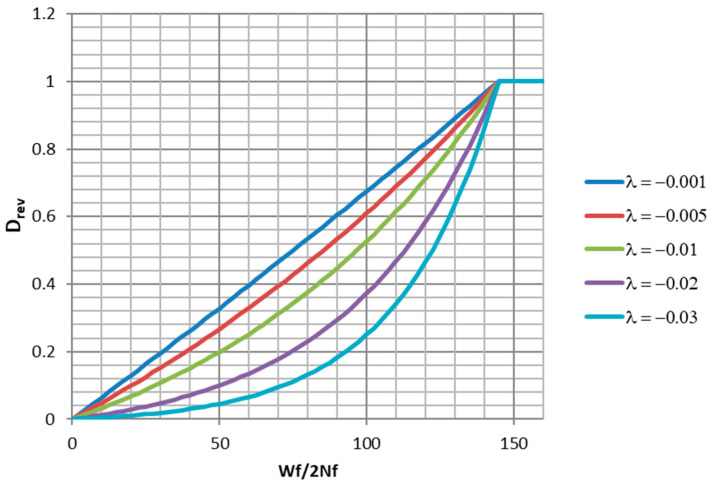
Plots of truncated exponential distribution with different shapes.

Although the trunated normal distribution has the best fit, the truncated exponential distribution has some desireable properties. If monotonic tension data points are available, they can be used to directly constrain the point where the curve is strictly equal to 1.0. The parameter λ controls the shape of the curve between x=0 and x=a. For λ close to zero, the curve is nearly a ramp function. For negative λ values, it has varying degrees of concave upwards curvature. Examples of a family of such curves are plotted in Figure 22. In the present case, λ=−0.0319, giving a strongly rising curve. A damage function of the mathematical form of Equation (42) exists in the literature [2]. The authors of [2] present Equation (43) as an improvement to the Coffin–Manson relationship (Equation (2)) for modeling LCF in the sub 100 cycle range ($\epsilon_{pa}$ is the plastic strain amplitude). The relationship is presented as an empirical improvement and is not derived from physical principles. The authors do not describe it as a truncated exponential distribution function. It is clear that Equation (43) can be rearranged to a form similar to Equation (42).

(43) $$D_{cycle}=\frac{Exp\left(\frac{\lambda \epsilon_{pa}}{\epsilon_{f}}\right)-1}{Exp\left(\lambda \right)-1}$$

## 5. Concluding Remarks

In this study, the Maximum Entropy principle was shown to provide a systematic theoretical and philosophical basis for selecting a CDF to model damage. The method was demonstrated on an LCF data set for aluminum 2024-T351, but the proposed approach is equally applicable to ductile metals undergoing fatigue damage. In general, the relationship between the measured plastic dissipation per cyclic reversal and the damage per reversal is nonlinear, suggesting that the total work of fracture or the total entropy to cause fracture varies with the loading condition. We showed that several maximum entropy distributions, including the truncated exponential and the truncated normal distribution, are good choices for material damage modeling. Compared to the exponential distribution, the truncated exponential distribution has additional flexibility and can model concave upwards trending data. In the limit, it can approximate a uniform distribution. For the aluminum 2024-T351 alloy, the truncated normal distribution was shown to provide the best fit to the data, relative to the more common alternatives of Coffin–Manson equation or the Weibull distribution. Left truncation of the normal distribution extends its applicability to the many applications where data is non-negative. Finally, a Coffin–Manson function in terms of plastic strain (the standard form) was compared to the truncated normal distribution and shown to provide an inferior fit.

## Figures and Tables

**Table 1:** Maximum entropy (MaxEnt) distributions corresponding to moment functions *g_r_*(*x*) [13,18,19,20].

Support	Type	*g_r_*(*x*)	Distribution Function	Reference
[a, b]	Discrete	N.A.	Uniform	[13]
[0, ∞)	Discrete	*x*	Exponential	[13]
[0, ∞)	Continuous	*x*	Exponential	[18]
[0, a]	Continuous	*x*	Truncated Exponential	[19]
[0, ∞)	Continuous	*x* ^2^	Half Normal	[20]
(−∞, ∞)	Continuous	*x*, *x*^2^	Normal	[18]
[0, ∞)	Continuous	*x*, *x*^2^	Left Truncated Normal	[20]
[0, a]	Continuous	*x*, *x*^2^	Left and Right Truncated Normal	[20]
[0, ∞)	Continuous	ln(*x*), *x^β^*	Weibull	[18]

**Table 2:** Low-cycle fatigue data summary [22].

*k*	2*N_f_*	Stress Amplitude MPa	Plastic Strain Amplitude	Data
1	1	537.810	0.2	Values
2	1	558.495	0.28	Values
3	76	503.335	0.01725	Values
4	38	495.061	0.0129	S-20 Loop fitted
5	124	492.993	0.0123	Values
6	144	482.650	0.012	Values
7	190	475.755	0.01067	Values [22]
8	114	477.824	0.0085	S-17 loop fitted
9	440	466.792	0.0083	Values [22]
10	560	448.175	0.00606	Values [22]
11	920	437.143	0.00472	Values [22]
12	516	453.691	0.0038	S-12 loop fitted
13	1080	441.280	0.0037	Values
14	800	441.280	0.0036	Values
15	624	454.381	0.0035	S-11 loop fitted
16	2800	398.531	0.00178	Values [22]
17	1608	430.938	0.0017	S-18 loop fitted
18	5860	403.358	0.0007	Values
19	16336	351.645	0.00015	Values
20	23400	358.540	0.00004	Values

**Table 3:** Inelastic dissipation and damage.

*k*	2*N_f_*	Range Mpa	Range Ep	*1/n*	*rho*	*W_f_*/2*N_f_*	*D_rev_*
1	1	538	2.00 × 10^−1^	26.7	0.964	1.04 × 10^2^	1.00
2	1	558	2.80 × 10^−1^	26.7	0.964	1.51 × 10^2^	1.00
3	76	1007	3.45 × 10^−2^	26.7	0.928	1.61 × 10^1^	1.32 × 10^−2^
4	38	990	2.58 × 10^−2^	26.7	0.928	1.19 × 10^1^	2.63 × 10^−2^
5	124	986	2.46 × 10^−2^	26.5	0.927	1.12 × 10^1^	8.06 × 10^−3^
6	144	965	2.40 × 10^−2^	26.4	0.927	1.07 × 10^1^	6.94 × 10^−3^
7	190	952	2.13 × 10^−2^	25.9	0.926	9.40	5.26 × 10^−3^
8	114	956	1.70 × 10^−2^	25.1	0.923	7.50	8.77 × 10^−3^
9	440	934	1.66 × 10^−2^	24.7	0.922	7.15	2.27 × 10^−3^
10	560	896	1.21 × 10^−2^	21.2	0.910	4.94	1.79 × 10^−3^
11	920	874	9.44 × 10^−3^	19.1	0.900	3.72	1.09 × 10^−3^
12	516	907	7.60 × 10^−3^	17.6	0.893	3.08	1.94 × 10^−3^
13	1080	883	7.40 × 10^−3^	17.4	0.891	2.91	9.26 × 10^−4^
14	800	883	7.20 × 10^−3^	17.1	0.890	2.83	1.25 × 10^−3^
15	624	909	7.00 × 10^−3^	16.9	0.888	2.83	1.60 × 10^−3^
16	2800	797	3.56 × 10^−3^	13.6	0.863	1.22	3.57 × 10^−4^
17	1608	862	3.40 × 10^−3^	13.4	0.862	1.26	6.22 × 10^−4^
18	5860	807	1.40 × 10^−3^	13.4	0.862	4.87 × 10^−1^	1.71 × 10^−4^
19	16336	703	3.00 × 10^−4^	13.4	0.862	9.09 × 10^−2^	6.12 × 10^−5^
20	23400	717	8.00 × 10^−5^	13.4	0.862	2.47 × 10^−2^	4.27 × 10^−5^

**Table 4:** Candidate function forms fit to data in Table 4.

Function	Form (for 0 ≤ *a*)	Sum of Sqr Error
Left Truncated Normal	$D_{rev}=\frac{F_{norm}\left(W_{c},\mu ,\sigma \right)-F_{norm}\left(0,\mu ,\sigma \right)}{1-F_{norm}\left(0,\mu ,\sigma \right)}$	5.17
Truncated Exponential	$D_{rev}=\frac{1-\exp\left(-\lambda W_{c}\right)}{1-\exp\left(-\lambda a\right)}$	5.45
Power law (Coffin–Manson form)	$D_{rev}=k{\left(W_{c}\right)}^{-\frac{1}{c}}\;for\;W_{c}\leq W_{c\;crit}$	14.8
Weibull	$D_{rev}=1-\exp\left(-kW_{c}{}^{\alpha }\right)$	15.4
Smith–Ferrante form	$D_{rev}=1-\left(1+kW_{c}\right)\exp\left(-kW_{c}\right)$	57.3

## Nomenclature

**Variable**	**Definition**
$\Delta \epsilon_{p}$	Plastic strain range
$\epsilon_{f}\prime$	Fatigue ductility coefficient
c	Fatigue ductility exponent
$N_{f}$	Total cycles (loops) to failure
D(t)	Material damage parameter as a function of time or pseudo-time
Δσ	Stress range—total height of stress–strain loop
$\Delta \epsilon_{total}$	Total elastic plus plastic strain range
$\sigma_{f}$	True fracture stress
$\epsilon_{f}$	True fracture strain
$W_{f}$	Total inelastic dissipation (per unit volume) to failure
$A_{f}$	Inelastic dissipation (per unit volume) per stress–strain loop area of stabilized loop
$2N_{f}$	Total reversals to failure
H	The entropy of a probability distribution
S	Gibbs entropy
$p_{i}$	Probability mass function value of the *i*th random state
$k_{b}$	Boltzmann’s constant
I(p)	The information associated with an event with probability *p*
$g_{i}\left(x\right)$	The *i*th moment function
$\lambda_{i}$	The Lagrange multiplier corresponding to the *i*th moment function
f(x)	The probability density function (PDF) of the random variable x
F(x)	The cumulative distribution function (CDF) of the random variable x
μ	Mean value of a random variable
σ	Standard deviation of a random variable
α	Weibull distribution shape parameter
β	Weibull distribution scale parameter
γ	Euler’s constant
K	Ramberg–Osgood strength parameter
$\frac{1}{n}$	Ramberg–Osgood exponent
